# Choices of chromatographic methods as stability indicating assays for pharmaceutical products: A review

**DOI:** 10.1016/j.heliyon.2021.e06553

**Published:** 2021-03-27

**Authors:** Yik-Ling Chew, Mei-Ann Khor, Yau-Yan Lim

**Affiliations:** a Faculty of Pharmaceutical Sciences, UCSI University, No. 1 Jalan Menara Gading, UCSI Heights, 56000, Cheras, Kuala Lumpur, Malaysia; b School of Science, Monash University Malaysia, Jalan Lagoon Selatan, Bandar Sunway, 47500 Petaling Jaya, Selangor Darul Ehsan, Malaysia

**Keywords:** Stability, Pharmaceutical, Degradation, Impurities, Forced degradation, Drug stability

## Abstract

Stability indicating assay describes a technique which is used to analyse the stability of drug substance or active pharmaceutical ingredient (API) in bulk drug and pharmaceutical products. Stability indicating assay must be properly validated as per ICH guidelines. The important components in a stability indicating assay include sensitivity, specificity, accuracy, reliability, reproducibility and robustness. A validated assay is able to measure the concentration changes of drug substance/API with time and make reliable estimation of the quantity of the degradation impurities. The drug substance is separated and resolved from the impurities. Pros and cons of HPLC, GC, HPTLC, CE and SFC were discussed and reviewed. Stability indicating assay may consist of the combination of chromatographic separation and spectroscopic detection techniques. Hyphenated system could demonstrate parallel quantitative and qualitative analysis of drug substances and impurities. Examples are HPLC-DAD, HPLC-FL, GC-MS, LC-MS and LC-NMR. The analytes in the samples are separated in the chromatography while the impurities are chemically characterised by the spectroscopy in the system. In this review, various chromatographic methods which had been employed as stability indicating assays for drug substance and pharmaceutical formulation were systematically reviewed, and the application of hyphenated techniques in impurities characterisation and identification were also discussed with supporting literatures.

## 1 Introduction

**Figure 1:**
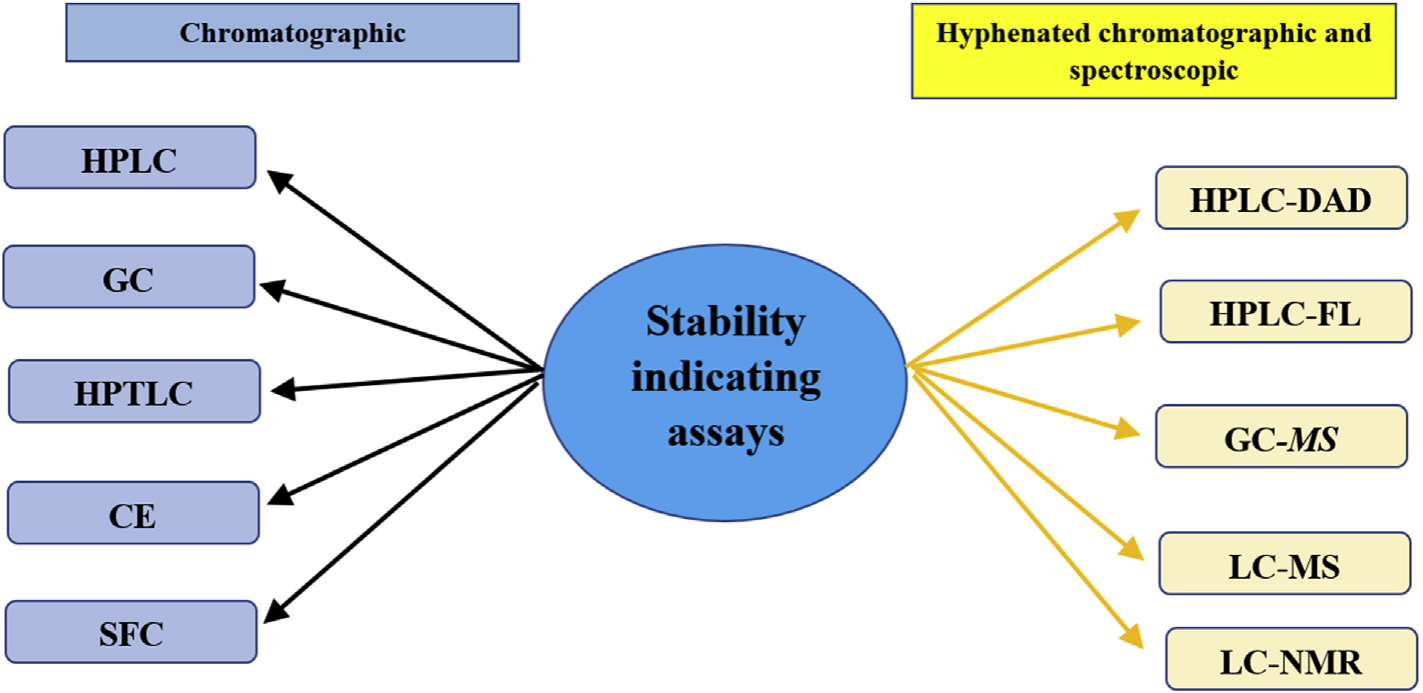
Stability indicating assays for pharmaceutical products.

Stability-indicating assay is utilised in the forced degradation analysis of pharmaceutical products and active pharmaceutical ingredients (Blessy et al., 2014). It is a procedure that could detect the degradation and change in active pharmaceutical ingredient (API) concentration in pharmaceutical products (Rawat and Pandey, 2015). United States Food and Drug Administration (FDA) guidance documents defined that stability indicating method is a validated quantitative analytical procedure that can be used to evaluate the stability of the drug substance (Blessy et al., 2014). It is also a method which could measure the changes in drug substance concentration without the interference from other substances present, including degradation impurities, excipients and other potential substances (U.S. Department of Health and Human Services, 2000). The International Council for Harmonisation of Technical Requirements for Pharmaceuticals for Human Use (ICH) guideline Q3B, Impurities in New Drug Products stated that it is mandatory to provide documented evidence, to show that the analytical methods are properly validated and they are suitable for detection and quantification of degradation products and impurities (Guideline, 2006). The validated methods should be reliable, specific and able to demonstrate that the impurities of the new drug substance are separated from the API and other pharmaceutical substances. Various methods have been implemented as stability indicating assay. The common ones include high performance liquid chromatography (HPLC), gas chromatography (GC), high performance thin layer chromatography (HPTLC), capillary electrophoresis (CE) and super critical fluid chromatography (SFC). Some of these chromatography methods could also be coupled with other spectroscopy methods as modern high-end and high-resolution separation and chemical characterisation techniques, e.g. high performance liquid chromatography-diode array detector (HPLC-DAD), high performance liquid chromatography-Fluorescence (HPLC-FL), gas chromatography-mass spectrometry (GC-MS), liquid chromatography-mass spectrometry (LC-MS) and liquid chromatography-nuclear magnetic resonance (LC-NMR) spectroscopy. With the chromatography-spectroscopy combination, many degradation impurities were identified and documented (Figure 1).

## 2 Stability indicating assays

There is no single assay or parameter that could profile the stability of all products. The suitability of the methods is dependent on the chemistry and physiochemical properties of the API and ingredients in the formulations. Therefore, knowledge of the physiochemical properties of the drug substance and the pharmaceutical formulations is extremely crucial. The properties of targeted substance such as pK_a_ value, log *P*, solubility, polarity, volatility and absorption maximum (λ_max_) of the drug must be known (Blessy et al., 2014). These physiochemical properties could provide important information on selection of stability indicating assays and the parameter settings. For instance, log *P* and solubility of API and formulations are taken into consideration in selection of mobile phase and sample solvent in HPLC, while pK_a_ values could determine the suitable pH for the mobile phase (Patel Riddhiben et al., 2011; Blessy et al., 2014). Understanding the chemical profile of APIs, such as the chemical structures, chemical properties, the degradation pathways, number of degradants, and the optimum conditions for peaks separation are equally important in the development of stability indicating assays (Jadhav et al., 2012). The mandatory information can be retrieved from scientific literatures, company drug profiles, spectral libraries and reports (Patel Riddhiben et al., 2011). Many studies have reported the use of various stability indicating assays in analysing the degradation of API and pharmaceutical products. The main objective of this review is to provide an overview on various stability indicating assays used in forced degradation studies, and the list of drugs which had been successfully analysed and their impurities were resolved using specific techniques.

### 2.1 High performance liquid chromatography (HPLC)

HPLC is the dominant technique in pharmaceutical analysis. HPLC is carried out in a chromatographic column in which a solid or liquid sample is dissolved in a suitable solvent. This system is simple to operate, versatile, requires minimal sample preparation, provides high resolution and excellent recovery (Ravisankar et al., 2017, Khan et al., 2020; Alsohaimi et al., 2018). This technique is also applicable for numerous types of compounds, such as compounds with diverse polarity, molecular mass, volatility and thermal sensitivity (Kumar and Kumar, 2012). Analyte elution could be performed either in isocratic or gradient elution mode (Table 1). The separation output of HPLC is represented in chromatogram and each analyte in the sample is displayed as a sharp peak at a specific time (Kazakevich and Lobrutto, 2007; Raza et al., 2015). HPLC is an extremely useful technique for drug stability evaluation. It is specific, rapid, sensitive, and robust (Ravisankar et al., 2017). The unique properties of HPLC include various detection wavelengths that can be set for detection; adjustable flow rate and the mobile phase elution profile (Aljerf and AlMasri, 2018).

**Table 1:** Stability indicating methods for drug substances and their elution conditions.

Analytical methods	Drug substances	Elution conditions	References
		Elution mode; Mobile phase	
High performance liquid chromatography (HPLC)	Ezetimibe	Gradient elution; ammonium acetate buffer (pH 7.0) and acetonitrile	(Singh et al., 2006)
	Losartan potassium and hydrochlorothiazide	Gradient elution; phosphate buffer solution of (pH 7.0), with acetonitrile	(Hertzog et al., 2002)
	Atorvastatin and amlodipine	Isocratic elution; acetonitrile-NaH_2_PO_4_ buffer (pH 4.5)	(Mohammadi et al., 2007)
	Docetaxel	Gradient elution; water-acetonitrile	(Rao et al., 2006)
	Glucosamine	Isocratic elution; acetonitrile-phosphate buffer (pH 7.5)	(Shao et al., 2004)
	Sacubitril and valsartan	Isocratic elution; acetonitrile-citrate buffer (pH 3)	(Ahmed et al., 2017)
	Sacubitril and valsartan	Isocratic elution; trifluoroacetic acid in water-methanol	(Jyothi and Umadevi, 2018)
	Sacubitril and valsartan	Isocratic elution; potassium phosphate buffer (pH 3.0)-methanol	(Patel et al., 2017), (Mishara et al., 2017)
	Sacubitril and valsartan	Isocratic elution; acetonitrile-methanol-potassium dihydrogen phosphate (pH 3.8)	(Naazneen and Sridevi, 2017)
	Sacubitril and valsartan	Isocratic elution; ammonium acetate buffer (pH 4)-acetonitrile	(Moussa et al., 2018)
	Vancomycin hydrochloride	Isocratic elution; buffer citrate (pH 4)-acetonitrile-methanol	(Serri et al., 2017)
	Curcumin	Isocratic elution; acetonitrile-methanol-water (pH 3)	(Ansari et al., 2005)
	Excedrin (acetaminophen, aspirin, and caffeine)	Gradient elution; trifluoracetic acid and mixture of trifluoracetic acid-methanol-acetonitrile	(Dongala et al., 2019)
	Flibanserin	Isocratic elution; ammonium acetate buffer (pH 3) and acetonitrile	(Chew et al., 2020)
	Enrofloxacin and piroxicam	Isocratic elution; acetonitrile and water (pH 3)	(Araujo et al., 2020)
Carrier gas			
Gas chromatography (GC)	Rosmarinic acid	Helium	(Razboršek, 2011)
	Divalproex sodium	Helium	(Subasranjan et al., 2010)
	Acetaminophen and aspirin	Nitrogen	(Bergh and Lötter, 1984)
	Magnesium valproate		(Ambasana et al., 2011)
	Memantine hydrochloride	Nitrogen	(Jadhav et al., 2012)
Mobile phase composition			
High performance thin layer chromatography (HPTLC)	Curcumin	Chloroform:methanol (9.25:0.75 v/v)	(Ansari et al., 2005)
	Pseudoephedrine and cetirizine	Ethyl acetate–methanol–ammonia (7:1.5:1, v/v/v)	(Makhija and Vavia, 2001)
	Trimetazidine	N-butanol-water-methanol-ammonia (20%) (14:0.2:0.2:2, v/v/v/v)	(Thoppil et al., 2001)
	Timolol maleate	Ethyl acetate–methanol–isopropyl alcohol–ammonia (25%) (80:20:2:1, v/v/v/v)	(Kulkarni and Amin, 2000)
	Piroxicam	Toluene–acetic acid (8:2 v/v)	(Puthli and Vavia, 2000)
	Ezetimibe and simvastatin	N-hexane–acetone 6:4 (v/v)	(Dixit et al., 2008)
	Estradiol	Chloroform–acetone–isopropyl alcohol–glacial acetic acid (9:1:0.4:0.1, v/v/v/v)	(Kotiyan and Vavia, 2000)
	Isoniazid and rifampicin	N-hexane–2-propanol–acetone–ammonia–formic acid, 3:3.8:2.8:0.3:0.1 (v/v)	(Ali et al., 2007)
	Aspirin and clopidogrel bisulphate	Carbon tetrachloride-acetone (6: 2.4 v/v).	(Damle et al., 2009)
	Dabigatran etexilate mesylate	Toluene: ethyl acetate: methanol: formic acid (3:4:3:0.2, v/v/v/v)	(Prajapati et al., 2017)
	Mangiferin	Ethyl acetate: ethanol: formic acid (10:1.5:1v/v/v)	(Padh et al., 2017)
	Empagliflozin and Linagliptin	Methanol: toluene: ethyl acetate (2: 4: 4v/v/v)	(Bhole et al., 2017)
	Saxagliptin	Toluene: methanol: ammonia (6:4:0.2 v/v/v)	(Ghode et al., 2019)
	Diphenhydramine	Ammonia: methanol: ethyl acetate (2.5 : 5: 42.5 v/v/v)	(Bober, 2017)
Column; background electrolyte (BGE)			
Capillary electrophoresis (CE)	Metformin hydrochloride	Fused silica capillaries; citrate buffer (40 mM, pH 6.7)	(Hamdan et al., 2010)
	Metformin hydrochloride, saxagliptin hydrochloride, and dapagliflozin	Deactivated fused silica capillary; phosphate buffer (30 mM, pH 6.0)	(Maher et al., 2019)
	Tramadol	Uncoated fused-silica capillary; borate buffer (50 mM, pH 10.2)	(Mohammadi et al., 2011)
	Amlodipine	Fused-silica capillary; phosphate running buffer (100 mM, pH 3.0)	(Fakhari et al., 2008)
	Buserelin	Bare fused silica capillary, phosphate buffer (pH = 3.00; 26.4 mM)	(Tamizi et al., 2014)
	gemifloxacin and lomefloxacin	Fused silica capillary; H_3_PO_4_–NaOH running buffer (25 mM; pH 8.5)	(Elbashir et al., 2008)
	Norfloxacin	Fused-silica capillary; phosphate (10 mM; pH 2.5)	(Alnajjar et al., 2007)
	Carvedilol and hydrochlorothiazide	Fused silica capillary; phosphate buffer (12.5 mM; pH 7.4)–methanol (95 + 5, v/v)	(Alzoman et al., 2013)
	Isradipine	Fused-silica uncoated capillary; borate buffer (15 mM; pH 9.3)	(Aguiar et al., 2011)
Column; elution mode; mobile phase			
Super critical fluid chromatography (SFC)	Clofarabine	Ethylene bridged hybrid 2-ethylpyridine (BEH 2-EP) column; isocratic elution; liquid CO_2_ and methanol (70:30 v/v)	(Ganipisetty et al., 2013)
	Mometasone furoate	Silica column; gradient elution; liquid CO2 and methanol (5–15% methanol)	(Wang et al., 2011)

HPLC could simultaneously detect various analytes in pharmaceutical formulations (Table 1). It has been vastly used as stability indicating assay for bulk drugs and drug products, separating drug substances and degradation impurities simultaneously. For instance, Dongala et al. (2019) recently had developed a stability indicating assay using HPLC to separate 14 impurities from Excedrin tablet which consisted of acetaminophen, aspirin, and caffeine via gradient elution. The separation was excellent, and the peaks were perfectly resolved in the chromatogram. The optimisation of the parameters to achieve good separation of multiple drugs could be achieved using the response surface methodology in HPLC where the retention time response the surfaces of the three drugs present in Excedrin tablet. The retention times of the three drugs would not be identical if the three surfaces did not intersect. The capacity factor of a good chromatography should be neither too low nor too high. The separation method in HPLC were developed based on the optimisation of mobile phase, including the concentration of organic modifier and pH. The pH of the mobile phase may affect the degrees of ionisation of analytes, the stationary phase and mobile phase additives. The selectivity and the analytes retention times change with pH.

In addition, Araujo et al. (2020) had successfully developed and validated the method for simultaneous determination of enrofloxacin and piroxicam and their respective degradation products in veterinary formulations. The method showed good specificity, high precision, accuracy, sensitivity, and robustness, which is suitable for routine quality-control analysis, as per ICH guidelines. Our recent studies have also managed to separate up to six degradation impurities in flibanserin in the stability indicating assay developed using HPLC (Chew et al., 2020).

It is interesting to observe HPLC had been continuously used in development of stability indicating studies of sacubitril and valsartan (Ahmed et al., 2017; Patel et al., 2017; Mishara et al., 2017; Naazneen and Sridevi, 2017; Jyothi and Umadevi, 2018; Moussa et al., 2018). Various combinations of mobile phases were used to achieve better separation for sacubitril, valsartan and their impurities. These studies utilised isocratic elution and weak acidic mobile phases which consist of buffers and organic solvents (Table 1). Many studies have reported that HPLC showed promising sensitivity, reliability, linearity, accuracy, precision, repeatability, robustness, limit of detection (LoD) and limit of quantification (LoQ) and it is extremely useful to be used as stability indicating method for various types of pharmaceutical ingredients and products (AlFaris et al., 2020a, 2020b, 2020c; Al Shamari et al., 2020; Shrivastava and Gupta, 2011). This is also the reason why HPLC is a popular technique used in drug stability evaluation (Ciobanu et al., 2017). The condition could be customised based on the physiochemical properties of drugs (Sinan Kaynak et al., 2017).

HPLC has several limitations. Since the mobile phase utilises organic solvents, hence this method can be expensive, and non-environment friendly as the organic wastes eluted from the HPLC system require proper waste disposal (Yabré et al., 2018). Besides, HPLC does not have a universal detector for stability testing. Although ultraviolet-visible (UV-vis) detector is commonly used for chromophoric compounds, there is no single detector that could detect all chemical compounds. HPLC may possibility have low sensitivity for certain compounds, and some may not be detected if these chemical species are irreversibly adsorbed to the HPLC packing materials (Nikolin et al., 2004). HPLC is easy to operate, but the operation may be time-consuming. To use HPLC as stability indicating method, five complementary steps are required: (1) selection of the type of methods, (2) gather the information of sample and analyte, (3) method development, (4) method optimisation, and (5) method validation (Dong, 2013; Michael et al., 2020).

### 2.2 Gas chromatography (GC)

Gas chromatography (GC) is a method that utilises gases to separate and analyse compounds that can be vaporised without decomposition. To analyse a sample using GC, the sample is dissolved in a solvent before it is injected into the system. The sample is vaporised before the analytes are separated between stationary and mobile phases. Chemically inert gas, such as helium and nitrogen, carries the analytes through the heated column, where the separation and partition of analytes happens. GC works similarly to HPLC and thin layer chromatography (TLC), except that it has liquid stationary phase and gaseous mobile phase.

GC has high precision, accuracy, sensitivity and resolution in sample analysis and peaks separation. It had been used as stability indicating assay for numerous pharmaceutical substances and products since 1980s. Bergh and Lötter (1984) developed the stability indicating assay for acetaminophen and aspirin. Subasranjan et al. (2010) developed a validated assay for divalproex sodium in pharmaceutical formulation. Both studies reported that the stability indicating assays were able to quantify the standard drugs, detect and resolve the degradation impurities and other substances or contaminants present in the pharmaceutical matrices. GC had been used for drug stability of magnesium valproate and other salt form of valproic acid. The detection and quantification the impurities were determined as per ICH guidelines (Ambasana et al., 2011). GC analysis is also applicable to non-chromophoric substances in drugs. It had been used in the detection of memantine hydrochloride and its non-chromophoric impurities in bulk drug and drug products, where they had been successfully resolved via GC system (Jadhav et al., 2012). Most of the stability studies using GC as the analytical technique commented that GC method is specific, accurate, linear, reproducible, rugged, and robust (Subasranjan et al., 2010).

GC is more environmentally friendly than HPLC because it minimises the environmental pollution and save organic solvents (Subasranjan et al., 2010). However, GC system is only limited to the analysis of volatile samples and samples with lower melting point (Sojitra et al., 2019). Chemical compounds with molecular weight above 1000 Da are difficult to vaporise because they are rarely volatile (Feng et al., 2019). Hence this method is more suitable for smaller size molecules. Even if the chemical species could vaporise, thermally unstable molecules are also not suitable for GC analysis (Feng et al., 2019). Besides, the sample to be analysed by GC must be salt free and absence of ions (de Koning et al., 2009).

### 2.3 High performance thin layer chromatography (HPTLC)

High performance thin layer chromatography (HPTLC) is the advanced version of TLC which provides better separation efficiency, and it is suitable for both qualitative and quantitative analysis (Choukaife and Aljerf, 2017). This method is rapid and cheap (Ansari et al., 2005), the results are reproducible and large number of samples could be analysed simultaneously with small amount of mobile phase. Combinations of organic solvents are also applicable in HPTLC as mobile phase (Table 1). The mobile phases can be mixture of non-polar and polar organic solvents (Ansari et al., 2005; Dixit et al., 2008; Damle et al., 2009; Bhole et al., 2017), as well as combination of organic with acidic or alkaline solvents (Makhija and Vavia, 2001; Thoppil et al., 2001; Kulkarni and Amin, 2000; Puthli and Vavia, 2000; Kotiyan and Vavia, 2000; Ali et al., 2007; Prajapati et al., 2017; Padh et al., 2017; Ghode et al., 2019). This method is also suitable for samples that require mobile phases with extreme pH, where the ionisation state of the analytes was dependent on the pH of the mobile phases (Mohammad and Moheman, 2011). Vast combination of mobile phase allows simultaneous separation of analytes in drug samples (Devanand et al., 2011). This method is especially suitable for samples that require combination of mobile phases as this is not achievable via other analytical methods, especially HPLC (Dhandhukia and Thakker, 2011). This system is also applicable to suspension samples. It produces coloured bands and retention factors for analytes identification (Loescher et al., 2014).

Numerous studies showed the specificity of HPTLC in drug stability analysis. For instance, Ansari et al. (2005) had employed HPTLC as stability indicating assay for analysis of curcumin in pharmaceutical formulation. The curcumin in pharmaceutical formulation was spotted on TLC aluminium plates precoated with silica gel 60F_254_ and the TLC plates were developed in chloroform:methanol (9.25:0.75 v/v) solvent system. The peaks of curcumin and degradants were analysed using densitometer with wavelength set at 430 nm (Gupta et al., 1999). This method was selective and exhibited high precision, specificity and accuracy in the stability studies of curcumin. This is in agreement with Bober (2017), who had reported the stability of diphenhydramine using HPLTC equipped with densitometer. Reduction in diphenhydramine content in the spot and appearance of degradation impurities peaks were noticed in the densitograms upon exposure to thermal and light stresses.

However, HPTLC has several limitations. The separation bed is short with limited developing distance and lower plate efficiency (Kamboj and Saluja, 2017). This limitation may result in ineffective separation if the retention factors, R_f_ values and the polarities of analytes are similar to each other where the spots and peaks of the analytes will overlap with each other (Aljerf et al., 2017). Sample derivatisation may be needed prior to detection, if it is not detectable under 254 nm, 336 nm and white light (Loescher et al., 2014).

### 2.4 Capillary electrophoresis (CE)

Capillary electrophoresis (CE) is a high performing separation method which is carried out in narrow-bore capillaries with the influence of external electric field (Al Azzam et al., 2011; Anastos et al., 2005). This method is applicable to various substances, including inorganic ions, chiral biomolecules, biotechnological, biopolymers and clinical samples (El Deeb et al., 2013; Anastos et al., 2005). Separation in CE is selective, highly precise and efficient. It is able to analyse complex mixtures, and requires small sample size (in microliter range or below) and reagents (El Deeb et al., 2013; Gordon et al., 1988; Anastos et al., 2005; Currell, 2008). CE has several advantages over HPLC and GC. CE method is versatile. The separation time is short and it is suitable for thermally unstable compounds (Anastos et al., 2005; Currell, 2008). CE can also be used to separate structurally similar compounds, i.e. chiral molecules. Compared with HPLC and gas chromatography, capillary electrophoresis has distinct advantages, including automation, minimal sample preparation, low cost of capillary columns, use of very small amounts of organic solvents and chemicals (Thormann et al., 1996).

CE has become a complementary and alternative method in stability indicating assay. It is feasible in separation of drugs and impurities which have similar structures and chemical properties in pharmaceutical formulations (Fakhari et al., 2008; Altria and Rogan, 1994; Alnajjar et al., 2007). The samples require either no or minimum pre-treatment (Cianciulli and Wätzig, 2012; El Deeb et al., 2013; Anastos et al., 2005) before analysis. CE system is also applicable to water insoluble, charged and neutral drug substances (Altria, 2013; Anastos et al., 2005). Therefore, the system is applicable to various pharmaceutical product analysis, including stability indicating studies, determination of drug impurities, main component assays, chiral separation and detection of drug residue (Altria, 2013). For instance, stability of metformin hydrochloride in tablet was evaluated using CE and the method developed showed good linearity, accuracy, precision, sensitivity, selectivity and robustness (Hamdan et al., 2010). Metformin hydrochloride was successfully resolved from its major degradation products in this study (Hamdan et al., 2010).

CE is able to resolve and differentiate enantiomers and structurally similar compounds with different polarity and solubility (Altria, 2013). Highly sensitive, selective and accurate nature of CE system is shown in the stability indicating assay of amlodipine under various stresses (Mohamed et al., 2016). Degradation was noticed under acid and alkaline hydrolysis, oxidative and photolysis. R-(+) and S-(-)-amlodipine enantiomers were detected as impurities upon degradation (Fakhari et al., 2008). The excipients in the tablet and the enantiomers were perfectly resolved and appeared as sharp peaks in electropherogram. CE was also used as stability indicating assay for tramadol (TR). Mohammadi et al. (2011) had developed a chiral stability-indicating assay using CE system to evaluate the stability of TR enantiomers. To assist in separation of the chiral molecule, maltodextrin was added into the buffer as chiral selector (Tabani et al., 2015). The studies showed that both (+)-TR, (-)-TR and the degradation impurities were detected as individual peaks in electropherogram.

The main limitation with CE as the stability indicating method is the separation of analytes with different polarity and water solubility (Toraño et al., 2019). This is seen in the stability study of quetiapine, an antipsychotic drug for the treatment of schizophrenia (Hillaert et al., 2004). Series of impurities were present in this drug, which were produced during synthesis, acid hydrolysis and oxidative degradation, namely desethanol quetiapine, N-formyl-quetiapine, quetiapine carboxylate, N-ethylpiperazinyl thiazepine, ethylquetiapine, bis(dithiazepine) (dimer), N- and S-oxides (Borst et al., 2013). Due to the variation in water solubility of these impurities, CE was not suitable for such analysis.

### 2.5 Super critical fluid chromatography (SFC)

Super critical fluid chromatography (SFC) functions similarly to GC and HPLC. It merges the advantages of GC and HPLC (Hofstetter et al., 2019; Hage, 2018), but it utilises supercritical fluids such as carbon dioxide (CO_2_) as the mobile phases. SFC can be connected to wide range of detectors, such as Flame Ionization Detector (FID), Flame Photometric Detector (FPD), Electron Capture Detector ECD, Mass Spectrometer (MS) and Fourier Transformer Infrared, fluorescence emission spectrometer, and thermionic detectors (Lafont et al., 2012; Pavan and Raja, 2020; Thiébaut, 2018; Jumhawan and Bamba, 2017). FID and MS are commonly used for SFC (Pavan and Raja, 2020). This method is sustainable and more cost effective. It is considered as green technology, because it uses less organic solvents, produces less system waste and eco-friendly (Ganipisetty et al., 2013).

The distinct physical properties of SFC have several advantages over conventional HPLC. The analysis is more rapid and its gas like mobile phase has lower viscosity and higher diffusion coefficients than HPLC (Berger, 2007; Pinkston, 2005, Pavan and Raja, 2020). SFC is more preferred for compounds with high solubility in organic solvents (Wang et al., 2011). This method is reliable, rapid, displayed good efficiency in separation and could separate analytes with different polarities. It allows higher flow rates and the system could utilise shorter or longer columns than conventional HPLC (Pinkston, 2005; Pavan and Raja, 2020). The solvent evaporation and product isolation using SFC system are also rapid (Montañés and Tallon, 2018). SFC also has better resolving power than HPLC due to the high diffusivity of the mobile phase, which could lead to better separation of the chemical species in shorter running time (Pavan and Raja, 2020). SFC also has several advantages over GC. It could analyse chemical species which is thermally unstable, high molecular weight and without the need of derivatization to convert polar groups into non polar (Pavan and Raja, 2020). These advantages would make SFC a better choice of chromatographic method for pharmaceutical substances with these properties.

SFC has excellent performance, cost effective and requires minimum use of solvent. SFC was used in profiling the impurities in API degradation (Alexander et al., 2013; Ganipisetty et al., 2013; Majewski et al., 2005). Ganipisetty et al. (2013) had effectively separated clofarabine and its impurities within 6 min with SFC. The validated assay was rapid, accurate, precise, specific, robust and showed good linearity. This method also provides orthogonal selectivity, which is complementary to RP-HPLC. This is also in agreement to Wang et al. (2011), where authors had quantified mometasone furoate and its impurities using SFC, and the results showed that it was comparable to RP-HPLC. Authors reported that the SFC method developed is suitable for stability testing for mometasone furoate, due to its good linearity, high accuracy and precision. Alexander et al. (2013) had critically evaluated the advantages and disadvantages of HPLC and SFC in impurity profiling of lamivudine, festinavir and efavirenz in pharmaceutical products. Both analytical methods possess their pros and cons.

Despite the advantages of SFC over HPLC and GC, SFC has one of the biggest limitations. It is not able to analyze extremely polar samples due to the nonpolar mobile phase (Silva and Collins, 2014). CO_2_ lacks of polarity and hence it may be quite challenging to elute polar compounds from the stationary phase (Pavan and Raja, 2020). To overcome this, a polar modifier, either methanol or ethanol will be added in small amount to increase the polarity. Higher temperatures and pressures will be required to increase the reactivity if too much modifier has been added. This will possess health risk to the operator. Comparison of the parameters in HPLC, HPTLC, GC, CE and SFC had been summarised in Table 2.

**Table 2:** Comparison of the various analytical methods on the basis of various parameters in pharmaceutical drugs analysis.

Analytical methods	Application in pharmaceutical drugs	Mobile phases	Sensitivity	Analyst's skills required	Cost	Environment friendly
High performance liquid chromatography (HPLC)	Compounds with diverse polarity, molecular mass, volatility and thermal sensitivity	Liquid	High to ultra-high	High to very high	Moderate to high	No
Gas chromatography (GC)	Volatile samples, compounds only, non-chromophoric substances	Gas	High to ultra-high	High to very high	Moderate	Yes
High performance thin layer chromatography (HPTLC)	Polar and non-polar compounds, suspension samples	Liquid	Moderate to ultra-high	High	Moderate	Yes
Capillary electrophoresis (CE)	Thermally unstable compounds, chiral molecules, water insoluble, charged and neutral drug substances	Liquid	High	High to very high	Moderate	Yes
Super critical fluid chromatography (SFC)	Compounds with high solubility in organic solvents, thermally unstable, high molecular weight	Gas	High	High to very high	Moderate	Yes

## 3 Combination of hyphenated chromatographic and spectroscopic technique

Chromatographic method separates the chemical components in a mixture while spectroscopy provides selective information for identification of unknowns using standards or library spectra (Patel et al., 2010). The combination of separation and spectroscopic detection techniques could demonstrate both quantitative and qualitative analysis of known drug compounds and unknown impurities in pharmaceutical matrices (Cortese et al., 2020). Therefore, the characterization of unknown impurities requires sensitive, selective and sophisticated spectroscopic methods that could provide comprehensive structural information. These hyphenated techniques offer excellent separation efficiency, on-line complementary spectroscopic library and structure-related information of the impurities within reaction mixtures (Patel et al., 2010). Hyphenated techniques range from the combination of separation-separation, separation-identification and identification-identification techniques (Phale and Korgaonkar, 2009). Hyphenated methods are applied in characterization of impurities in forced degradation studies particularly when impurities cannot be isolated in pure form. Below are the examples of method which are commonly selected in impurities identification in drug stability indicating assay.

### 3.1 HPLC-photodiode array ultraviolet detector (HPLC-DAD)

**Figure 2:**
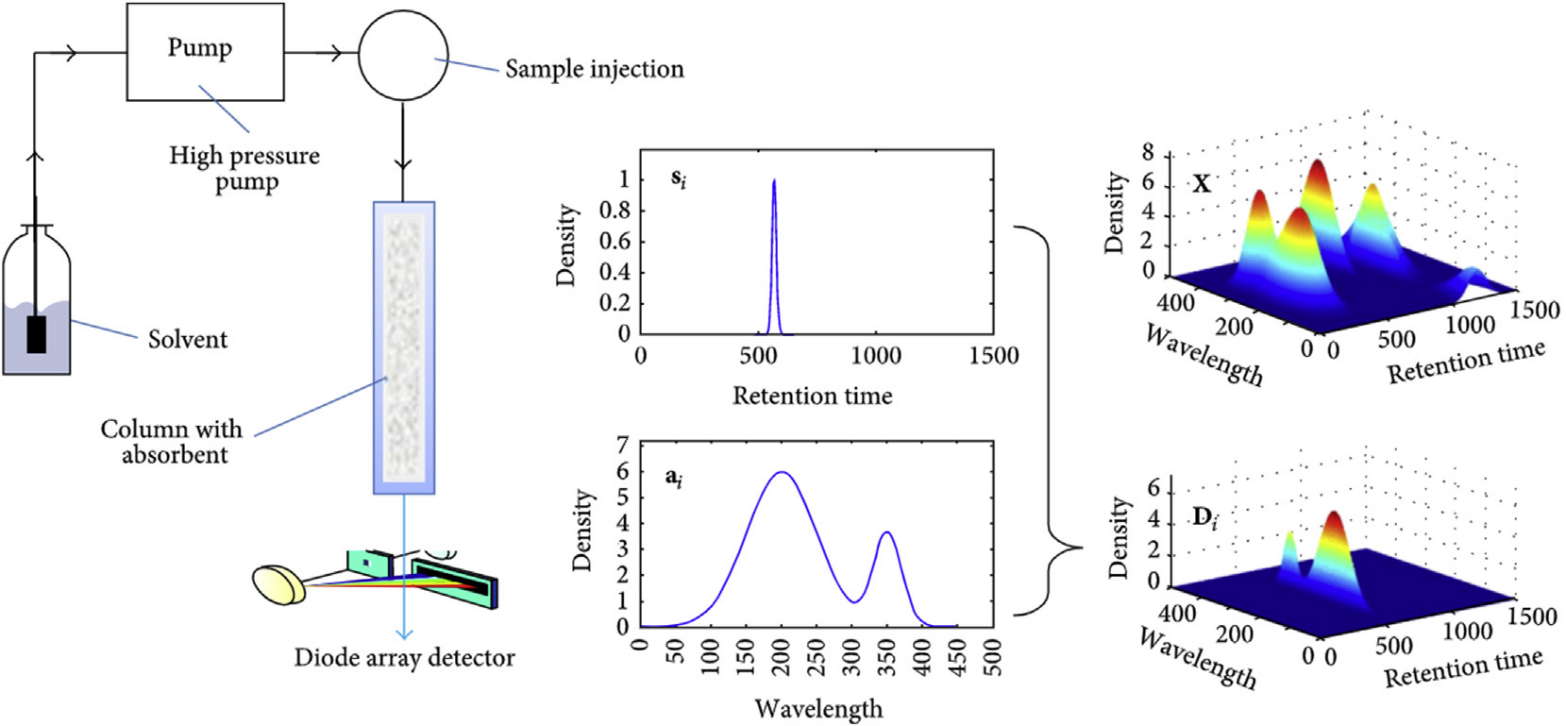
The principle of the HPLC-DAD data set. Reproduced from Cui et al. (2014). Figure reproduced is under Creative Commons Attribution License.

High Performance Liquid Chromatography coupled with Diode Array Detector (HPLC-DAD) could separate analytes into peaks in chromatogram and acquire spectra for all compounds in the ultraviolet and visible (UV-Vis) region of the spectrum (Cui et al., 2014; Patel et al., 2019; Ahmed et al., 2016). DAD acquires the spectra of peaks across a range of wavelengths simultaneously. Assessment of spectral peak purity and identification of unknown compounds could be performed (Figure 2).

Studies reported that HPLC-DAD is simple, specific, reliable and suitable to be used for routine analysis, quality control and stability indicating assay in pharmaceutical preparation (Baker et al., 2017; Shaalan et al., 2017; Sharma and Pancholi, 2010; Verbeken et al., 2011). HPLC-DAD provides diagnostic information about the drug substances and degradation impurities. Shifting in maximum absorption (λ_max_) could offer valuable information about the structural changes that take place in the degradation process. The degradation impurities of anti-malarial drug lumefantrine was identified using HPLC-DAD, where desbenzylketo derivative had been identified as degradant (Verbeken et al., 2011). DAD-UV spectra showed that λ_max_ of desbenzylketo impurities and lumafentrine were 266 nm and 234 nm, respectively. Shifting in λ_max_ showed the replacement of benzyl group of lumefantrine via keto function (Verbeken et al., 2011). Besides, Sharma and Pancholi (2010) had successfully identified the degradation impurities of olmesartan medoxomil using HPLC-DAD. UV spectra of olmesartan medoxomil was compared to the impurities. It was noticed that the λ_max_ 260 nm of ester moiety of drug substance was not visible in the spectra of impurities. Therefore, authors had deduced that the ester moiety, 5-methyl-2-oxo-1,3-dioxolen-4-yl-methyl group of olmesartan medoxomil had been de-esterified in the degradation process (Sharma and Pancholi, 2010).

Besides peak identification, DAD could also verify peak purity, where co-elution of other compounds, such as adjuvants and excipients could be detected. This technique was used as stability indicating assay for amlodipine besylate, valsartan and hydrochlorothiazide in antihypertensive mixtures (Shaalan et al., 2017) and dihydrochloride in hepatitis C antiviral agent (Baker et al., 2017). Both studies commented that DAD could identify and verify the drugs and impurities peaks. The purity angle of the peaks indicated the spectra homogeneity, where the obtained purity angles within the purity threshold limits would confirm the peaks were homogeneous and pure in samples in forced degradation (Baker et al., 2017).

### 3.2 HPLC- fluorescence detector (HPLC-FL)

HPLC- fluorescence detector (HPLC-FL) is a highly sensitive and specific method in detecting fluorescent analytes. The light emission from analyte could be detected and measured by Fl detector. It is useful in detecting analytes with natural fluorescence. When the light energy is absorbed by the analyte, some of the electrons would be raised to excited state. When the electrons had returned to the ground state, fluorescence light would be emitted. The FL detector is coupled to the HPLC for detection. This method had been used in the analysis of pharmaceuticals and clinical samples, especially with samples with high levels of impurities.

HPLC-FL was reported to have high sensitivity, selectivity, and repeatability. Kamal et al. (2019) reported that HPLC-FL showed that it is better than HPLC-DAD as the stability indicating method was equally simple, accurate, and reproducible for the analysis of daclatasvir bulk drug and drug products. They had made comparison between HPLC-FL with Ultra-High Performance Liquid Chromatography (UPLC) coupled with DAD detector. The sensitivity of the detection was enhanced using FL detector. The stability indicating method of cyproheptadine hydrochloride, a sedating antihistamine drug had also been developed and compared to the United States Pharmacopeia (USP) method (Sharaf El-Din et al., 2018). It was reported that the HPLC-FL method developed was comparable to the USP method in terms of reliability, sensitivity, accuracy, precision specificity, and robustness. HPLC-FL is also useful for simultaneous determination of more than one drugs from plasma samples. Sacubitril and valsartan from rat plasma were determined using HPLC-FL. It was reported that both analytes were determined simultaneously from the plasma sample and the study showed good linearity and correlation coefficient. The percentage recovery, relative standard deviation and relative error were within the acceptable range (Attimarad et al., 2018). It was proposed that this method was suitable to be used in pharmacokinetic studies of clinical samples, because the sample preparation is simple and the analysis time is short.

However, FL detectors are not commonly available compared to DAD detector. In addition, the addition of a fluorescence derivative is needed if the analytes do not fluoresce naturally.

### 3.3 Gas chromatography-mass spectroscopy (GC-MS)

Gas chromatography-mass spectroscopy (GC-MS) is a direct, fast, and reliable method for the separation, quantification and identification of drug and impurities in forced degradation studies. GC-MS uses the energetic electron to ionise and fragment analyte molecules before mass spectrometric analysis and detection. Molecular fingerprint or fragmentation pattern of the analyte will then be compared to the spectra library for compound identification. This method is specifically for analytes which could be resolved in GC.

A GC column is connected via a transfer device to a mass spectrometer. Samples to be analysed using GC-MS will first be separated in GC column, where the analytes are volatized. Analytes will pass through the MS ion source, where they will be impacted by the ionising electrons, causing the formation of cation radicals which are later fragmented into molecular ions. GC-MS is popularly used in drug and impurities analysis because it has comprehensive mass spectral library and its mass spectra is reproducible even in different instrument (Lynch, 2017). Furthermore, samples pre-treatment or derivatisation may not be required prior to analysis (Belal et al., 2009).

GC-MS had been used to resolve and identify the impurities in trimetazine dihydrochloride (Belal et al., 2014). The sample analysis was simple without sample pre-treatment and derivatization. The identity of the degradation impurities was revealed and confirmed via the fragmentation patterns in MS. The molecular fingerprint confirmed the structures of 2,3,4-trimethoxybenzyl alcohol and 2,3,4-trimethoxybenzaldehyde as the impurities.

GC-MS has also been used in identification of products of isomerisation (Aljerf and AlHamwi, 2018). It was used as the stability indicating assay to evaluate the stability of rosmarinic acid under various stresses, namely light, thermal, solvent and relative humidity (Razboršek, 2011). Razboršek (2011) reported that reduction of the *trans*-isomer peak was noticed in GC chromatogram, which indicated the isomerisation of *trans*-rosmarinic acid in the degradation process. The *trans*-isomer had slowly isomerised to the *cis*-form, where the *cis*-rosmarinic acid peak was increasing over time in the chromatogram. They had also noticed that the MS fragmentation pattern of the degradant was almost identical to the spectra of *trans*-rosmarinic acid. Therefore, the MS spectra of degradant was compared to the standard MS library and literatures to confirm the identification of *cis*-rosmarinic acid. Razboršek (2011) commented that this method is fast, specific, selective, accurate, precise and displayed satisfactory analytical performance of the method (i.e. LoD, LoQ, linearity, robustness).

### 3.4 Liquid chromatography-mass spectroscopy (LC-MS)

HPLC is widely used as stability indicating method in forced degradation studies. However, the results of HPLC analysis alone may not always be sufficient to elucidate and confirm the identity of the known and unknown degradation impurities (Marin and Barbas, 2004). HPLC method used in process analysis, impurity profiling, or stability studies is transferable to liquid chromatography-mass spectroscopy (LC-MS). LC-MS is applied for structural identification and confirmation. This technique is popularly used in characterization of degradation and drug impurities (Ramesh et al., 2014). LC-MS is a versatile tool which could separate and provide the information on the molecular weight and fragmentation pattern of the analytes. Based on the fragmentation pattern, reasonable chemical structures could be proposed (Qiu and Norwood, 2007).

LC separation utilises buffers and additives in mobile phases. The pH of the mobile phases can be controlled to ensure the ionisation of analyte. However, only LC-MS compatible modifiers such as formic acid, acetic acid, ammonium formate, ammonium acetate, ammonium bicarbonate, ammonium hydroxide, and volatile ion-pair reagents such as trifluoroacetic acid and hexafluorobutyric acid can be used (Qiu and Norwood, 2007; Garcia, 2005). The usage of non-volatile buffers and mobile phases in LC-MS system, such as phosphate, sulfate, borate, citrate, and octane sulfonate will cause deposition of salts on the ion source, resulted in capillary obstruction, suppress the ionization, affect the sensitivity and accuracy in analysis, and hence reducing the operation lifetime (Qiu and Norwood, 2007; Garcia, 2005).

LC-MS had been applied as stability indicating assays in numerous studies (Tolić et al., 2018). Some studies utilised HPLC in method development followed by LC-MS for compound identification. HPLC and LC-MS were selected in forced degradation impurities profiling due to its high precision, accuracy, specificity, selectivity, resolution and capacity (Marin and Barbas, 2004; Ramesh et al., 2014; Ramisetti and Kuntamukkala, 2014; Bhardwaj and Singh, 2008; Siddiqui et al., 2014, 2018; Wabaidur et al., 2013, 2015; Hakami et al., 2020). High temperatures and gases source were reported to result in higher sensitivity due to the ionic evaporation (Khan et al., 2016, 2020; Wabaidur et al., 2016). For instance, Marin and Barbas (2004) analysed acetaminophen, phenylephrine or phenylpropanolamine hydrochloride, chloropheniramine maleate and the degradation impurities in cough-cold products using validated HPLC method, followed by impurity profiling using LC-MS. Similarly, Bhardwaj and Singh (2008) analysed the stability of enalapril maleate using HPLC, followed by impurities characterisation in LC-MS. The application of LC-MS managed to resolve the structures of degradation impurities in numerous forced degradation studies (Marin and Barbas, 2004; Ramesh et al., 2014; Ramisetti and Kuntamukkala, 2014; Bhardwaj and Singh, 2008).

### 3.5 Liquid chromatography-nuclear magnetic resonance (LC-NMR)

**Figure 3:**
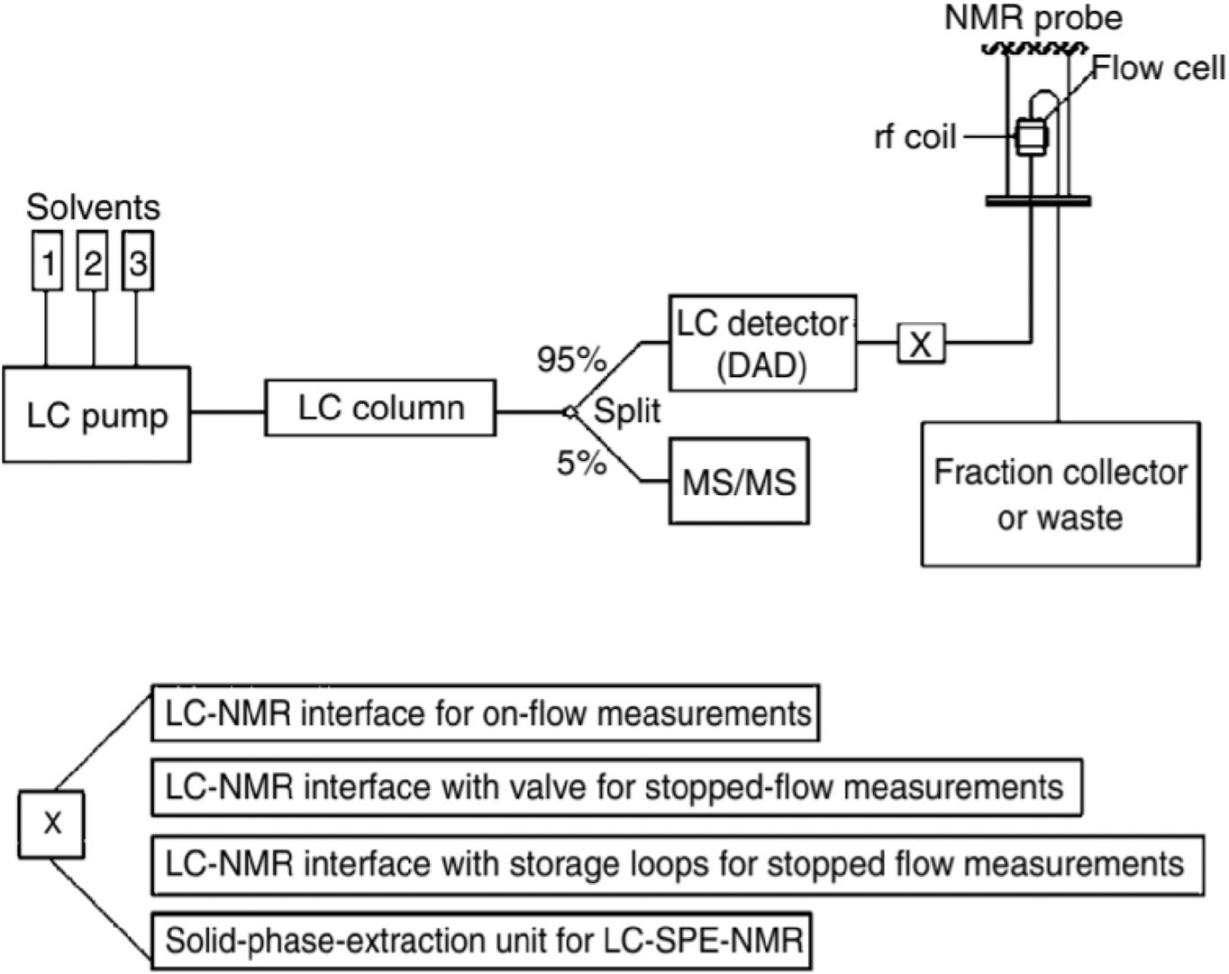
Operation mode of LC-NMR. Reproduced from Exarchou et al. (2005). Figure reproduced with permission from John Wiley & Sons.

LC-NMR is also a hyphenated technique which could be used to separate and characterize the degradation impurities in forced degradation studies. LC-NMR consists of various modes of operation (Figure 3), namely on-flow measurements, LC-NMR under static conditions, LC–NMR/MS and LC-solid-phase-extraction-NMR (Exarchou et al., 2005). Similar to the application of LC-MS, HPLC is normally used in method development and separation, followed by LC-NMR for structural characterisation. However, the cost of LC-NMR analysis is higher than LC-MS as deuterated solvents are required in the analysis (Elipe, 2011). Therefore, this technique is preferred if the impurities could not be isolated individually for structural characterisation (Singh et al., 2019).

LC-NMR had been used in degradant characterisation of various drug stability studies, such as n-hydroxy-1,3-di-[4-ethoxybenzenesulphonyl]-5,5-dimethyl-[1,3]cyclohexyldiazine-2-carboxamide (Peng et al., 1999), SCH 56592 (Feng et al., 2001), irbesartan (Shah et al., 2010), rosuvastatin (Shah et al., 2013), cilazapril (Narayanam et al., 2015) and fosamprenavir (Singh et al., 2019). However, the chemical characterisation using LC-NMR alone may not be sufficient to uncover the chemical structure of the unknowns. Therefore, other spectroscopy methods i.e. time-of-flight mass spectrometers (TOF MS), multi-stage mass studies (MS^*n*^) and online H/D exchange data, and others could also be used to determine the mass fragmentation pattern of the compounds and to support the structure elucidation. The combination of various spectroscopy methods had successfully determined the drug stability and characterised the impurities of irbesartan (Shah et al., 2010), rosuvastatin (Shah et al., 2013) and cilazapril (Narayanam et al., 2015).

## 4 Conclusion

Forced degradation studies provide information and knowledge about possible degradation mechanisms and the impurities formed in the degradation of the pharmaceutical API and help to elucidate the structure of the degradants. Stability indicating assay is mandatory in all forced degradation studies. However, no single stability indicating assay could fit perfectly into all drug stability studies. The selection and suitability of the technique is dependent on the chemical properties of the drugs and the impurities. Stability indicating assays developed should be validated for linearity, accuracy, sensitivity, precision, robustness, LoD and LoQ, as per ICH guidelines. A good stability indicating assay must be able to detect the stability and changes of drug substances and products with time, accurately measure the changes in API concentration without interference from other substances, including degradants, pharmaceutical impurities and excipients.

## Declarations

### Author contribution statement

All authors listed have significantly contributed to the development and the writing of this article.

### Funding statement

This work was supported by UCSI University Pioneer Scientist Incentive Fund (PSIF) research grant (Grant no.: Proj-In-FPS-012).

### Data availability statement

Data included in article/supplementary material/referenced in article.

### Declaration of interests statement

The authors declare no conflict of interest.

### Additional information

No additional information is available for this paper.
